# ‘On the Spot’ Digital Pathology of Breast Cancer Based on Single-Cell Mass Spectrometry Imaging

**DOI:** 10.1021/acs.analchem.1c05238

**Published:** 2022-04-12

**Authors:** Eva Cuypers, Britt S. R. Claes, Rianne Biemans, Natasja G. Lieuwes, Kristine Glunde, Ludwig Dubois, Ron M. A. Heeren

**Affiliations:** † Maastricht MultiModal Molecular Imaging Institute (M4i), Division of Imaging Mass Spectrometry, University of Maastricht, Universiteitssingel 50, 6229 ER Maastricht, The Netherlands; ‡ The M-Lab, Department of Precision Medicine, GROW—School for Oncology, University of Maastricht, Universiteitssingel 50, 6229 ER Maastricht, The Netherlands; § Russell H. Morgan Department of Radiology and Radiological Science, Division of Cancer Imaging Research, The Johns Hopkins University School of Medicine, Baltimore, Maryland 21205, United States; ∥ The Sidney Kimmel Comprehensive Cancer Center, The Johns Hopkins University School of Medicine, Baltimore, Maryland 21205, United States

## Abstract

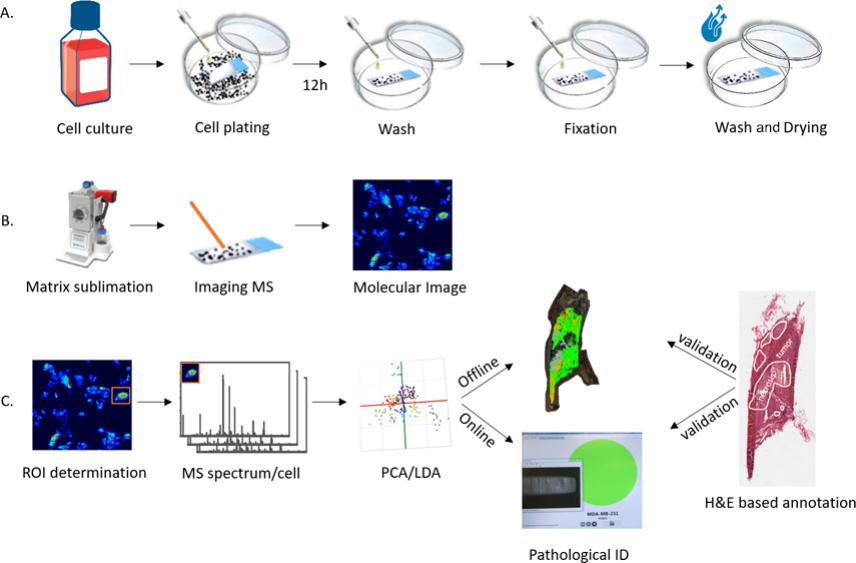

The molecular pathology of breast cancer is challenging due to the complex heterogeneity of cellular subtypes. The ability to directly identify and visualize cell subtype distribution at the single-cell level within a tissue section enables precise and rapid diagnosis and prognosis. Here, we applied mass spectrometry imaging (MSI) to acquire and visualize the molecular profiles at the single-cell and subcellular levels of 14 different breast cancer cell lines. We built a molecular library of genetically well-characterized cell lines. Multistep processing, including deep learning, resulted in a breast cancer subtype, the cancer’s hormone status, and a genotypic recognition model based on metabolic phenotypes with cross-validation rates of up to 97%. Moreover, we applied our single-cell-based recognition models to complex tissue samples, identifying cell subtypes in tissue context within seconds during measurement. These data demonstrate “on the spot” digital pathology at the single-cell level using MSI, and they provide a framework for fast and accurate high spatial resolution diagnostics and prognostics.

## Introduction

In breast cancer, genomic and transcriptomic heterogeneity has been extensively described.^1,2^ In addition, the breast tumor microenvironment can vary, resulting in tumor subtypes that are associated with distinct clinical behaviors^3,4^ and used as prognostic markers. For example, oestrogen receptor (ER) and progesterone receptor (PR) expression serve as predictors of hormone therapy responses^5,6^ and provide information on response to chemotherapy: ER– tumors respond better than ER+ tumors. Similarly, human epidermal growth factor receptor 2 (HER2) overexpression and/or gene amplification predict response to anti-HER2-targeted therapy. HER2 also provides prognostic information and can be used to help with diagnosis (i.e., Paget’s disease).^7^ Today, immunohistochemistry (IHC) is used as a standard to test these protein expression levels, despite the following major disadvantages: it is nonautomated, time-consuming, subject to human error, and relies on (subjective) pathologist interpretations. Furthermore, stains are not standardized worldwide, and different fixation times and methods lead to pre-analytical variability lacking robust internal controls.^7^ Furthermore, IHC is generally applied to proteins, and insights into the function and mechanism of lipid molecules and their role in the diagnosis and prognosis of breast cancer are steadily increasing.^8^

Eiriksson et al. showed that the heterogeneity of breast cancer subtypes is reflected in the expression levels of enzymes in lipid metabolism and, as a consequence, of lipid levels and ratios.^9^ This rich and valuable molecular information on lipid levels and ratios is currently neglected in routine pathological analysis because of technical, analytical, and interpretation challenges. Several alternatives to traditional IHC are available. The assessment of ER/PR/HER2 status based on mRNA expression can provide more objective, quantitative, and reproducible test results. However, mRNA analysis is challenging to apply to fixed tissue and very time-consuming. A newer and chemically information-rich approach for digital pathology on breast cancer that is described as midinfrared spectroscopic imaging. It was shown to offer label-free molecular recording and virtual staining by probing the fundamental vibrational modes of molecular components.^10,11^

Digital pathology, which includes scanning tissue slides and automating their analysis, offers many advantages over manual, analogue microscopic examination of glass slides alone. Digital images have improved the overall analysis, reduced the number of errors, and provided better contextual views of the tissue under study.^12^ Advances in machine learning have enabled the synergy of artificial intelligence and digital pathology, which in theory offers image-based diagnosis possibilities.^13^ All of these innovative approaches depend on information-rich images, rich in spatial, spectral, or molecular detail. Digital pathology applied to breast cancer has been limited by complications and challenges posed by its disease heterogeneity as described above.

Thus, the in-depth unraveling of the molecular differences, including lipids and their ratios, of breast cancer subtypes and developing an online subtype recognition method are of great prognostic and therapeutic interest and value. To interrogate whether transcriptomic differences are reflected in the local lipidome of breast cancer subtypes, we visualized with subcellular resolution the molecular profile distributions of 14 different in vitro cultured breast cancer lines with matrix-assisted laser desorption/ionization-mass spectrometry imaging (MALDI-MSI), in a mass range of *m*/*z* 200–1200, representing different metabolite and lipid abundances. We built recognition models based on these data and tested their ability to distinguish the different cell lines. Our recognition models successfully identified the breast cancer subtype on a single-cell level in breast cancer xenograft tissue sections, demonstrating its diagnostic applicability. As an ultimate proof of concept, we performed “on-the-fly” cell typing while scanning MDA-MB-231 tumor xenograft tissue sections.

## Materials and Methods

### Cell Preparation on Slides

Thirteen different breast cancer cell lines (full list in Supporting Information Table S1) were purchased from the Leibniz Institute DSMZ (Germany); a 14th cell line, MCF-7, was purchased from LGC Standards (Germany). All the lines were cultured in growth medium as indicated in Supporting Information Table S1. Indium tin oxide (ITO, CG-40IN-S115, Delta Technologies, USA) glass slides were coated with poly-l-lysine (20 μL of 1:1 dilution in water). Slides were washed with water before being placed in a 60 mm Petri dish with the conductive side facing up. Approximately 10^6^ cells (∼1.5 × 10^6^ cells/mL) were added to the Petri dish and incubated overnight at 37 °C with 5% CO_2_. Media was removed and slides were washed twice with phosphate-buffered saline. Neutral-buffered formalin (10%) was added for 10 min. Slides were washed twice with 50 mM ammonium formate and twice with Millipore water and dried under a gentle nitrogen stream.

For the cell pellets, the same cell culture was collected, centrifuged, and diluted 1/1 with a norharmane matrix (80 mg in 2 mL of MeOH). This solution was spotted on a Bruker target plate and evaporated under the nitrogen stream before loading into the timsTOF flex. The mean of five MALDI-1 spectra was taken by shooting directly on the cell pellet with the same instrument settings and laser intensity as used for the MSI experiments.

### Breast Cancer Xenograft Models

All the animal experiments were performed with appropriate ethical approval (2014-108 at GROW Maastricht University and A3272-01 at the Johns Hopkins University) and in compliance with the respective institutional guidelines. To generate tumor xenografts, 1.0 × 10^6^ MDA-MB-231 cells were resuspended in 50 μL of Matrigel basement membrane matrix (BD Biosciences, USA) and injected orthotopically into the mammary fat pad of female Crl:NU-Foxn1nu mice. When tumors were palpable, tumor volume was assessed by measuring the tumor in three dimensions using a vernier caliper and using the formula *a* × *b* × *c* × π/6, where *a*, *b*, and *c* are orthogonal diameters of the tumor, each corrected for the thickness of the skin (0.5 mm). At a tumor volume of ca. 200–500 mm^3^, tumors were excised and snap frozen. Xenografts were embedded in gelatin, then stored at −80 °C before sectioning.

### Sample Preparation for MSI

Tissue sectioning (12 μm, at −20 °C) was performed on fresh-frozen tissues using a Leica CM1860 UV cryotome (Wetzlar, Germany). Slides with tissue sections and cells were handled according to the same protocol: samples were kept at −80 °C prior to analysis. Beforemass spectrometry imaging (MSI), sublimation of 80 mg of norharmane at 140 °C for 180 s was performed using an HTX sublimator (HTX Technologies, USA). The sample preparation of tumor xenografts was the same for offline and online recognition.

### TimsTOF fleX (MALDI-1-MSI and MALDI-2-MSI)

Unless otherwise noted, MALDI, MALDI-2, and ion mobility MSI were performed on the timsTOF fleX MALDI-2 (Bruker Daltonics, Germany) in the positive ion mode with 50 laser shots per pixel and an interlaser pulse delay of 10 μs. Transfer settings were 350 V peak-to-peak (Vpp; funnel 1 RF), 400 Vpp (funnel 2 RF), and 600 Vpp (multipole RF). Focus pre-time-of-flight (TOF) transfer time was set at 90 μs and pre-pulse storage at 10 μs. The quadrupole ion energy was 5.0 eV with a low mass of *m*/*z* 300. Collision cell energy was 10.0 eV with collision RF at 200 Vpp. All the spectra were recorded using a 1 kHz laser repetition rate with 250 laser shots accumulated at each pixel. The average acquisition rate was 20 pixels per second over an *m*/*z* range between 200 and 1200 using a 5 × 5 μm^2^ pixel size for cells and a 30 × 30 μm^2^ pixel size for tissue analysis. Calibration of the instrument was carried out prior to every measurement with red phosphorus.

### Synapt

A Waters Synapt G2-Si HDMS system equipped with a prototype uMALDI source and provided with a Nd:YAG laser (Waters Corporation, UK) was used for online recognition experiments. For more detailed information about the uMALDI source, see Barré et al.^23^ Data acquisition was performed using MassLynx version 4.1 and HDImaging version 1.5 software (Waters Corporation). For online recognition, our model was built using the AMX Model Builder, which was loaded into AMX recognition software that was coupled to the data acquisition file. All the measurements were performed in the sensitivity mode with a scan rate of 1.0 s per scan, trap collision energy (CE) of 4, and transfer CE of 2, 1000 Hz laser repetition rate, and mass range of *m*/*z* 300–1200 in the positive ion mode. The instrument was calibrated with red phosphorus for the positive ion mode before each measurement. The spatial resolution was 30 × 30 μm^2^.

### Orbitrap Elite

Data were acquired via data-dependent acquisition (DDA) using a MALDI/ESI injector (Spectroglyph LLC, USA) coupled to an Orbitrap Elite hybrid ion trap-orbitrap mass spectrometer (Thermo Fisher Scientific GmbH, Germany). The MS1 data was acquired at a nominal mass resolution of 240,000 (full width at half maximum @ *m*/*z* 400) across *m*/*z* 200–1300, while MS/MS data was acquired in parallel using the ion trap with an isolation width of 1 Da, an activation Q of 0.170, and a normalized CE of 30 (manufacturer units).

### RapifleX

A Bruker RapifleX MALDI Tissuetyper TOF instrument equipped with a smartbeam laser (Nd:YAG, 355 nm) operating at 5000 Hz with 500 laser shots accumulated at each pixel was employed for MALDI-MSI. MALDI analyses were performed in the reflector positive ion mode in the mass range of *m*/*z* 200–1200 at a sample rate of 1.25 GS/s. Calibration was carried out in the positive ion mode using red phosphorus. The instrument was used at a 5 × 5 μm^2^ pixel size.

### Data and Statistical Analysis

After acquisition, data were imported and analyzed using MassLynx version 4.1 and HDImaging version 1.5 software for Synapt data (Waters Corporation), SCiLS Lab MVS for Rapiflex and timsTOF flex data (Version 2020b Premium 3D, build 8.01.12082, Bruker Daltonics), FlexImaging for Rapiflex and timsTOF flex data (Version 5, Bruker), XCalibur for Orbitrap Elite data (version 4.2.28.14, Thermo Scientific), and LipostarMSI for all the acquired data (version 1.10b17, Molecular Horizon). Figures were prepared using Abstract Model Builder (AMX, version 0.9.2092.0 [beta], Waters), SCiLS Lab, mMass (5.5.0, www.mmass.org), and Office 2016 software (Microsoft). The LIPID MAPS Structure Database (http://lipidmaps.org) and the ALEX123 lipid database (http://alex123.info/ALEX123/MS.php) were employed for molecular identification. Full details, including the lipid identification workflow, are described in Ellis et al.^19^

For offline recognition (i.e., post-data acquisition), a model was built using SCiLS Lab. Here, cells were randomly assigned to a training set and a validation set (two-thirds and one-third of the cells, respectively). For online recognition (i.e., during acquisition), AMX Model Builder and recognition software (Waters, v1.1.1966.0) were used. In order to evaluate the classification rate, the AMX model building data set was divided into five partitions (fivefold), each of which contains a representative proportion of each class within it (stratified). Four partitions (80%) of the data set were used to build a model under the same conditions as the original model. This model was used to predict the classifications of the one partition (20%) of the training set that was left out. 20% of the samples were left out. For cross-validation, outliers were defined based on the standard deviation with multiplier 3.

### Staining

Hematoxylin and eosin (H&E) staining was performed on the same sections used for MALDI-MSI experiments. Following MALDI experiments, ITO slides were first dipped in a 70% EtOH solution for 5 min in order to remove the residual matrix. H&E staining was subsequently conducted. Briefly, slides were hydrated in water for 1 min, followed by hematoxylin staining for 3 min, washed under running tap water for 3 min, stained with eosin for 30 s, and washed under running tap water for 3 min. Slides were then immersed in 100% EtOH for 1 min, transferred to xylene for 2 min, carefully covered with a coverslip, and dried at room temperature. The optical images were acquired at high resolution using the Leica AperioCS2 scanner with Aperio ImageScope (version 12.4.3.5008) software (Leica Biosystems Imaging, Germany).

## Results and Discussion

### Single-Cell MSI to Digital Pathology Strategy

**Figure 1:**
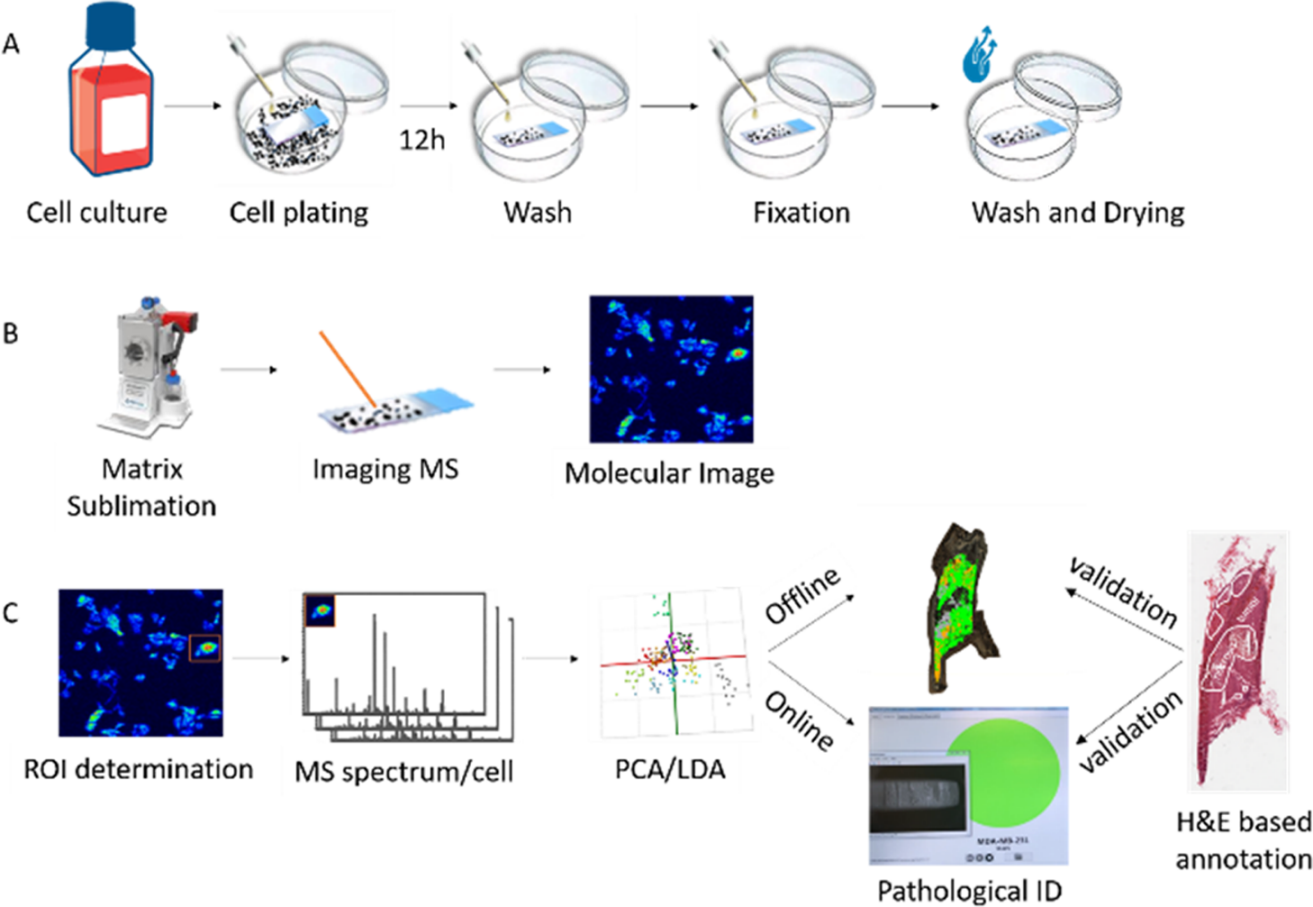
Experimental workflow from cell preparation to digital pathology. (A) Cell preparation on poly-l-lysine-coated ITO slides. (B) Sample sublimation and MSI analysis. (C) Data analysis encompassing ROI selection for every single cell, determination of the mean mass spectrum of every cell after rms normalization, model building using PCA/LDA analysis, and applying the generated method offline and online for pathological identification. H&E-based staining is considered the gold standard to which results should be compared.

We sought to design a robust analytical workflow from cell and tissue preparation to clinically relevant digital pathology and interpretation. To be applied to patient tissue, our workflow must successfully address the following criteria. First, a robust single-cell culturing and a MALDI-MSI sample preparation method that maintains single-cell molecular and spatial information needs to be developed. Second, the MALDI-MSI method needs to be able to acquire molecular data with subcellular spatial resolution combined with high sensitivity to detect lipids and metabolites within a single cell. Third, statistical analyses to discover and characterize cell subtype differences need to be implemented in a subtype recognition system. This recognition system needs to be clinically relevant and directly applicable to MALDI-MSI data of patient tissue, preferably independent of the MALDI-MSI equipment used. Fourth, ideally, this recognition method can be applied directly during a pathological imaging run, so fast and “on the spot” diagnostics become possible. An overview of the full experimental workflow is shown in Figure 1.

What are clinically relevant cell subtypes for digital pathology and interpretation? We selected 14 different breast cancer cell lines (Table S1) that represent three major breast cancer subtypes according to the status of ER, PR, HER2–HER2+, ER+PR+, and triple negative—which are considered to be predictors of therapy response and survival prognosis of a patient. Patients with hormone receptor-positive tumors often clinically benefit from receiving hormonal therapies that target the ER signaling pathway.^14,15^ Triple-negative tumor types (ER–, PR–, and HER2−) are described as the most aggressive, with the lowest survival rate.^15−17^ Because it was previously described that lipid expression patterns are directly linked to estrogen receptor expression rates,^18^ we selected these 14 lines in order to interrogate whether these different subtypes harbored different molecular profiles including lipids and their ratios. If statistical analysis of these molecular profiles is found to be subtype specific, we can implement them in a subtype recognition system and test whether it can be used for direct subtype identification in the context of the breast tissue environment. Altogether, our assay will allow direct spatial subtype detection according to the receptor status, meaning fast and automatic diagnostics and prognostics without any (immunohistochemically) staining process.

### Development of MSI Assay for Cultured Single Cells

**Figure 2:**
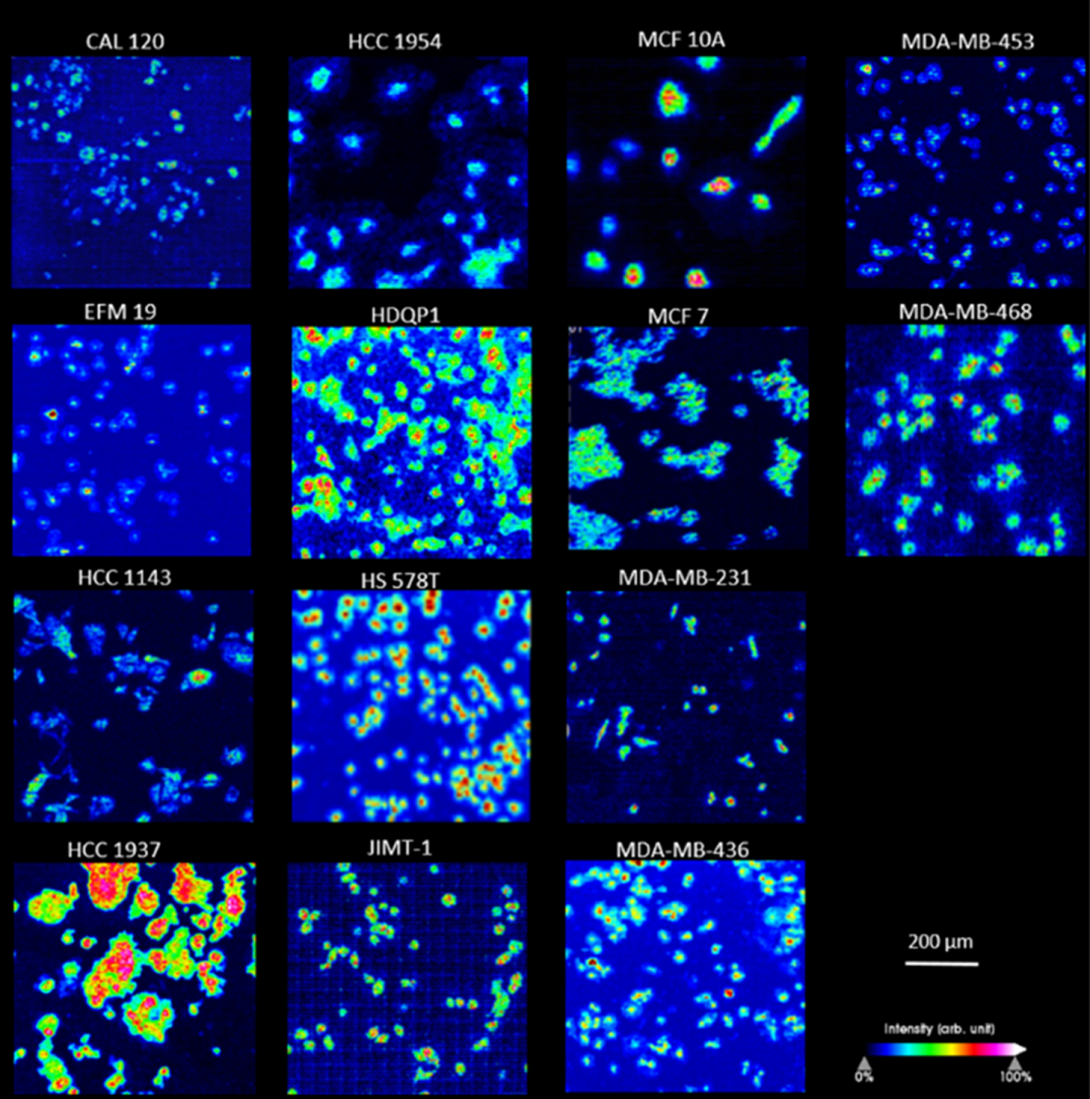
Example of the spatial distribution of PC 34:1 for all 14 analyzed cell subtypes after rms normalization. The scale bar represents 200 μm. Measurements were performed using timsTOF fleX MALDI-2 with a 5 μm pixel size.

Individual cells of the selected 14 human breast cancer cell lines (Supporting Information Table S1) were prepared on poly-l-lysine-coated ITO slides. MALDI- and MALDI-2-MSI data were acquired in the positive mode (Figure 2) to study the molecular composition of single breast cancer cells. The repeatability of the method was tested by comparing the molecular information of different cell cultures (*n* = 2) (Figure S2) and different ITO sample plates of the same cell line (*n* = 3). Moreover, the same samples were repeatedly measured on different days to rule out day-to-day variation. Before and after imaging, cell distribution, density, and shape were checked using light microscopy. All single cell types had a diameter of between 20 and 150 μm. A 5 × 5 μm^2^ pixel size allowed the acquisition of a minimum of four spectra per single cell. This high spatial resolution made it possible to visualize the intracellular distribution of compounds (Figure S1). Full single-cell spectra were analyzed by manually assigning the region of interest (ROI) for every cell and determining the mean spectrum of this ROI after root mean square (rms) normalization. For every slide, the highest-intensity cells were selected for incorporation into the database and recognition model. For every cell line, three different slides were measured on different days; in every measurement, 3–5 individual cells were randomly selected as the ROI. We were able to acquire a total of 229 single-cell spectra (ROIs) of the 14 different cell lines based on timsTOF fleX MALDI-2 data. Altogether, our assay allows to robustly image at subcellular resolution and determine the molecular profile of cultured single cells.

Why do we specifically want to use single-cell MSI in order to develop a molecular library? Indeed, it takes much more time and effort to perform our developed single-cell MSI method, and one could state that the same molecular (not spatial) information can be gathered from cell pellets. Because mean single-cell spectra will be used in molecular databases and recognition models anyway, it might save time and effort to directly use the molecular cell pellet profiles from pure cell lines. To prove that molecular profiles obtained from cell pellets are not the same as profiles obtained with single-cell MSI, we compared profiles from both (MSI and pellet) for two different cell types (MDA-MB-231 and HCC 1143). For every comparison, we used the same cell culture batch and the same instrument settings as used for the MSI experiments. As shown in Figure S3, lipid ratios clearly differ when mean spectra are taken from MSI or directly acquired from cell pellets.

### Lipid Identification at the Single-Cell Level

**Figure 3:**
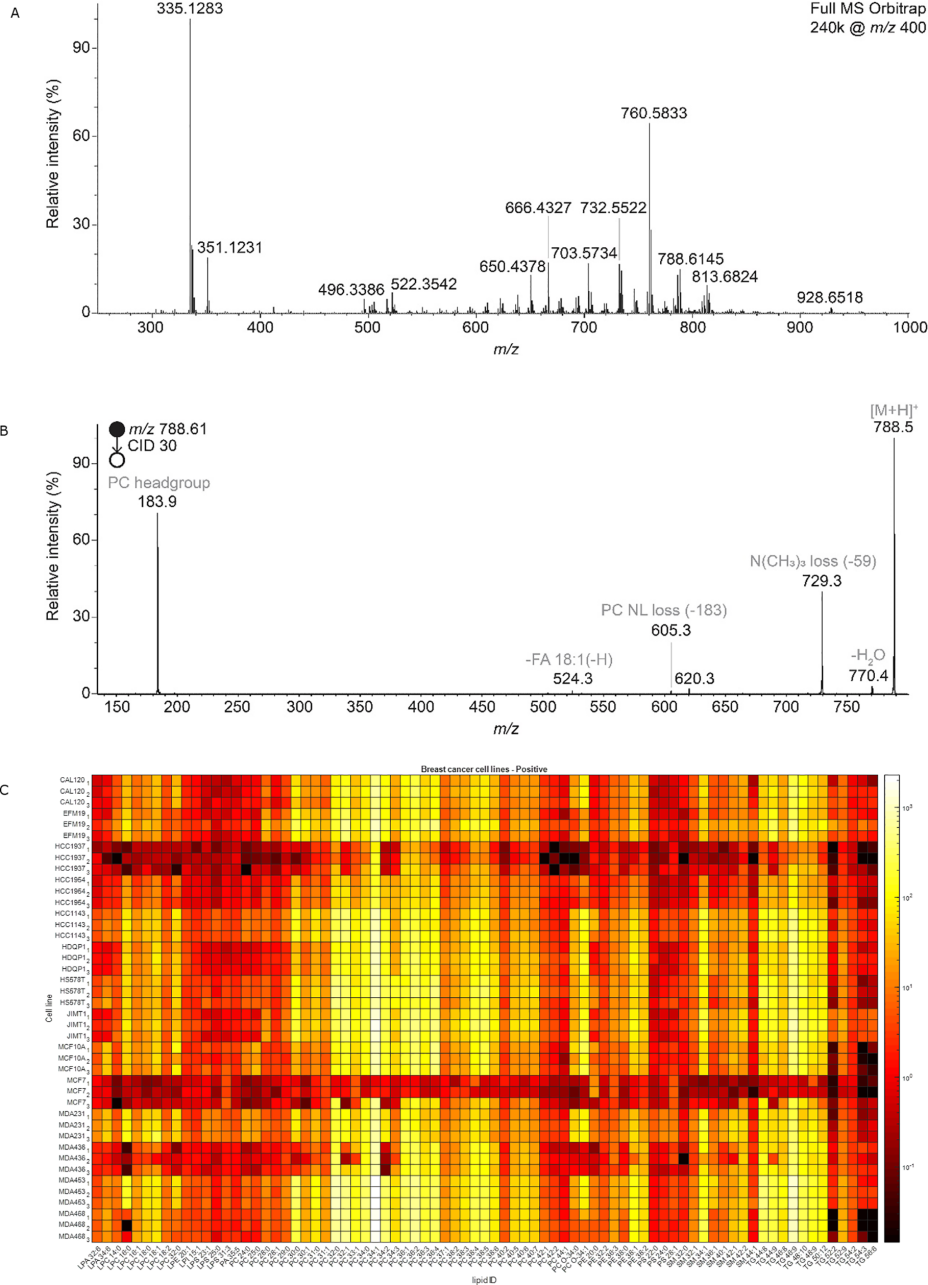
DDA identification of lipids measured on Orbitrap Elite. (A) Sample full MS spectrum in the positive ion mode and (B) MS2 of *m*/*z* 788.61 (PC 36:1), both measured in MDA-MB-231 cells. (C) Heat map of 79 identified lipids based on DDA analysis of single cells from the 14 breast cancer cell lines. Lipids identified are shown for three representative individual cells from three different cell cultures of the same cell line.

In the next step, we want to compare our acquired single-cell molecular profiles with earlier published findings and determine their relevance according to receptor expression rates and cell subtypes. Because it was previously described that lipid expression patterns are directly linked to estrogen receptor expression rates,^18^ we performed an automated, parallel MSI and structural identification of lipids using the Orbitrap-Elite-DDA.^19^ These measurements were acquired (Figure 3A,B) on the same slides as used in the imaging experiments and allowed the identification of 79 lipids present in all 14 cell lines (Figure 3C). Crucial to the success of this DDA approach is a sufficiently large number of cellular pixels being present in the image. We acquired a minimum of 10 (MS) and 8 (MS/MS) pixel scans per identified mass in a single data set to increase identification confidence. The identities were consistent with earlier described lipids found in cell pellet extracts analyzed with ultrahigh performance liquid chromotagraphy-quadrapole TOF-MS^9^ and in MSI data on MDA-MB-231 xenografts.^20^ For example, TG C-46 was found to be upregulated in MCF-7 (ER+/PR+/HER2−) and has low abundancies in MDA-MB (triple negative) subtypes. PC 30:1 and SM 42:2 are significantly more present in MCF-7 (ER+/PR+/HER2−) compared to MCF-10A (triple negative). The PC 42 series is highly abundant in MDA-MB-231 (triple negative).

The heatmap of the 79 identified lipids (Figure 3C) indicates that every cell line has a specific lipid profile, meaning different ratios of the same identified lipids, that allows genetically different breast cancer cells to be distinguished. Moreover, our data shows that these lipid profiles are robust within different cultures of the same cell subtype. These robust and unique lipid profiles linked to genetically different breast cancer cells are very promising for cell typing purposes. In the next step, we investigate if these differentiating profiles can be incorporated into recognition models for tissue cell identification.

### Recognition Models

Single-cell, MSI-based molecular models require two crucial elements to come together: (1) subcellular level spatial resolution with a minimum of 3 pixels per cell, meaning that for cells of 20 μm in diameter, we need a pixel size of 5 μm and (2) high sensitivity to acquire as much molecular information as possible in a broad *m*/*z* range. At the moment, these two requirements are best achieved by using timsTOF fleX MALDI-2. Indeed, it was described that timsTOF fleX MALDI-2 technology increases the sensitivity by approximately 3 orders of magnitude.^21−23^

In order to investigate whether our generated single-cell molecular profiles can be used for cell identification, we built recognition models based on the 229 single-cell timsTOF fleX MALDI-2 mass spectra between *m*/*z* 600–950. An AMX model was built for online (during measurement) recognition and a SCiLS Lab model for offline (post measurement) recognition, both using the same single-cell data.

**Figure 4:**
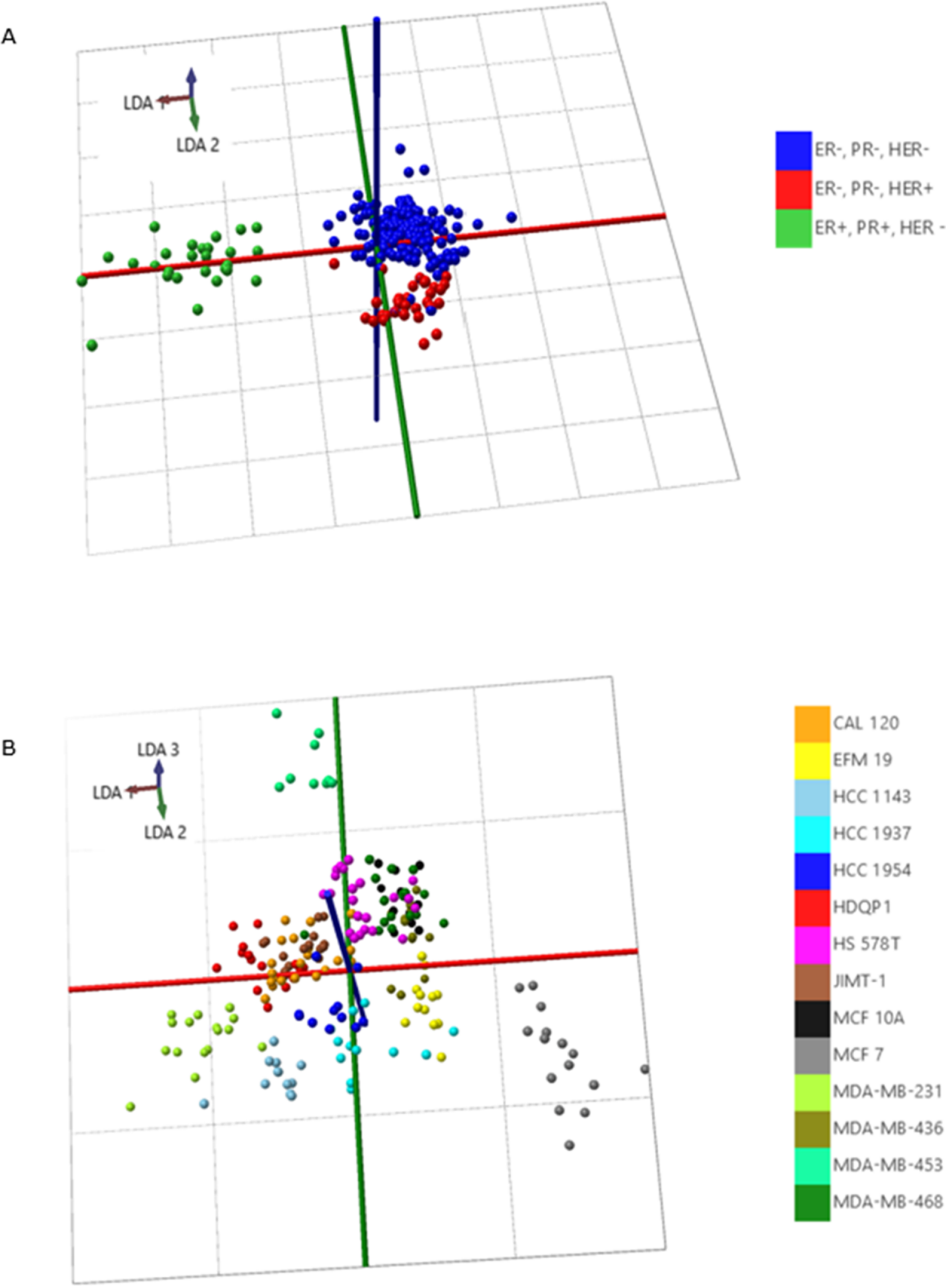
Classification models in AMX Model Builder (Waters) of (A) different molecular breast cancer subtypes based on ER, PR, and HER2 status and (B) different human breast cancer cell lines, based on single-cell profiles obtained from timsTOF fleX imaging experiments.

Using these models, we were able to separate the three breast cancer subtypes (triple negative, HER2+, and ER+PR+) with principal component analysis/linear discriminant analysis (PCA/LDA) analysis (Figure 4A). This model had a classification rate of 93.98% (excluding outliers) and 88.65% (including outliers) (Figure S4A), indicating that it could be a valuable diagnostic or therapy-response prognosis tool in breast cancer.

We also investigated whether we could make an even more detailed recognition model that is able to differentiate between the 14 cell subtypes. Thus, we acquired 229 single-cell spectra using timsTOF fleX MALDI-2 in the positive ion mode (Figure 4B) and built a classification model. A mass range of *m*/*z* 600–950 was also used with a binning of *m*/*z* 0.2. Cross-validation with 20% out and a standard deviation of 3 showed excellent classification performance, at 97.55% excluding outliers and 86.90% including outliers (Figure S4B). Based on the mass and loading plots of the recognition model and after DDA analysis, the 10 most prominent differentiating *m*/*z* values were all identified as lipids. Plots of single-cell intensities of these 10 lipids confirmed a specific profile for each cell line investigated in our study (Figure S5).

These results show that the acquired single-cell molecular profiles are indeed unique and specific for the different cell subtypes of breast cancer, with excellent cross-validation classification results. The last steps toward the clinical application of these single-cell molecular profiles and related recognition models are to investigate whether (1) these unique and distinguishing profiles are robust and independent across instrumentation platforms and (2) the same cellular profiles are also relevant in the complex tissue environment.

### Comparative Analysis of Single-Cell Mass Spectrometry Profiles on Different MALDI-MSI Instruments

**Figure 5:**
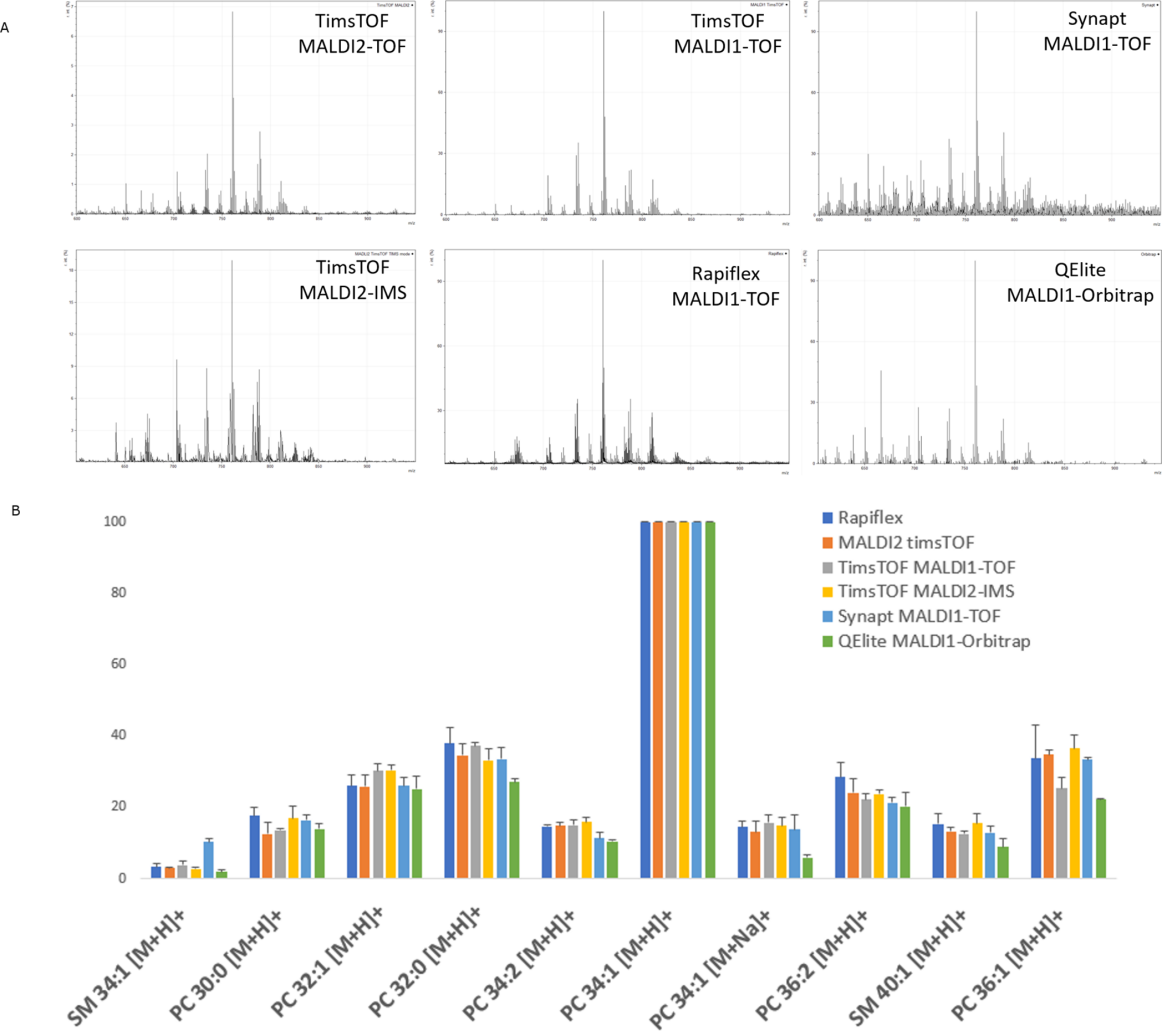
Comparison of mean normalized mass profiles of 10 differentiating lipids of single MDA-MB-231 cells after rms normalization measured on six different MSI instruments (positive mode). Error bars represent standard deviations from three randomly chosen cell profiles.

Cell profiles must be independent of the MALDI instrument used to be able to broadly deploy the recognition model as a standard diagnostic and prognostic tool. We thus investigated if the acquired single-cell profiles are robust across instrumentation platforms including MALDI instruments with different spatial resolutions and/or sensitivities. To evaluate this, the same samples were measured on timsTOF fleX MALDI-1 mode (Bruker Daltonics), timsTOF fleX TIMS mode (Bruker Daltonics), Rapiflex (Bruker Daltonics), MALDI-LTQ Orbitrap Elite (Thermo Fisher Scientific), and Synapt G2-Si HDMS (Waters). Single-cell spectra from the MDA-MB-231 line (*n* = 3) acquired on different instruments were normalized to the most abundant differentiating lipid (PC 34:1). The obtained ratios of the 10 most differentiating lipids were comparable on all six MALDI instruments (Figure 5).

Statistical analysis of these ratios using single factor analysis of variance showed *F* values below the critical *F* value of 3.105, confirming that there is no significant difference between the lipid profiles acquired on the different MALDI instruments. Moreover, the trend line clearly indicates a comparable pattern of these lipids for the different MALDI systems. This result indicates that the model, based on the timsTOF fleX MALDI-2 TOF spectra, is applicable to other MALDI-MSI instruments tested. In practice, this means our recognition models go beyond individual marker patterns, recognizing individual cell types based on full mass profiles, independent of the MALDI instrument used. Together, our analytical workflow, acquiring single-cell molecular profiles and translating them to cell subtype recognition models, can be applied to any MALDI-MSI instrument. Moreover, it is expected that this workflow is translatable to other cell subtypes, opening up new and fast diagnostic and prognostic tools in cell research.

### Spatial Analysis of Cell Subtypes in the Tissue Environment

**Figure 6:**
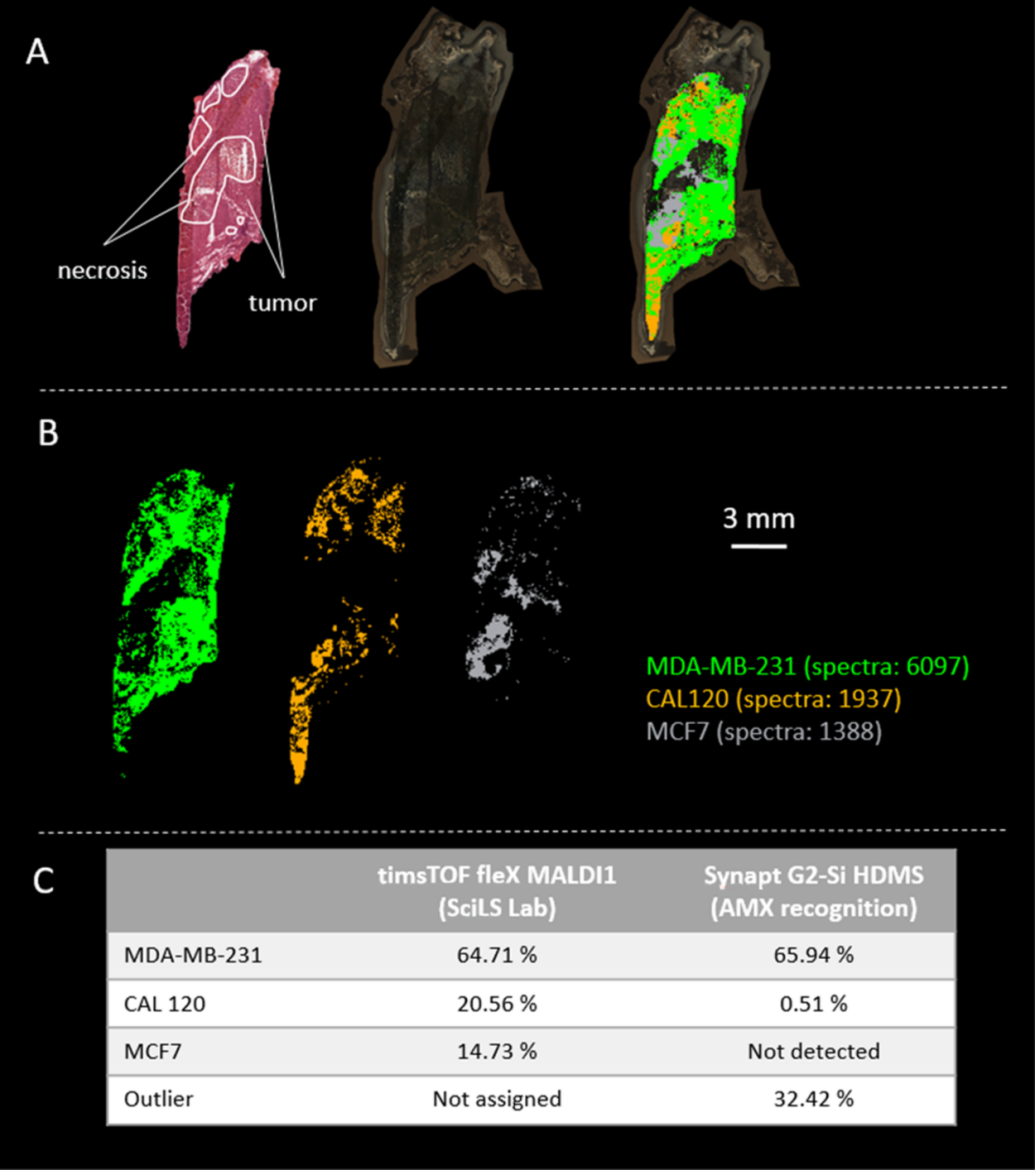
Automatic recognition of human breast tumor MDA-MB-231 xenografts from timsTOF fleX MALDI-1. (A) Annotated H&E staining (left), optical image of section (middle), and the distribution of identified cells combined with the overlayed optical image (right). (B) Separated images showing the distribution of the identified cells. (C) Comparison of cell recognitions measured on timsTOF fleX MALDI-1 and Synapt G2-Si HDMS. Percentages are calculated as the percentage of pixels classified (100% is the full measured region).

Having found that the lipid profiles were consistent across six different MALDI-MSI setups, we tested whether they could be used for complex tissue samples, a requirement for clinical applications. To do so, we assessed the model on MDA-MB-231 breast tumor xenograft samples and compared it with H&E staining-based annotation. Using timsTOF fleX, tumor tissue samples were measured with 30 × 30 μm^2^ spatial resolution. The acquired imaging data were post-processed using the cell subtype recognition model in SCiLS Lab, and showed that the main cell subtype was identified correctly (Figure 6). Two other cell lines (CAL120 and MCF-7) were recognized, probably a consequence of the typical properties of the SCiLS Lab recognition system, which forces all the data points into one of the 14 cell classes of the model rather than classifying them as outliers (such as necrotic tissue, background signal, gelatin, etc.). H&E staining confirmed that MCF-7 and CAL120 classified regions partly correspond to necrotic regions.

The same MALDI-MSI data were also post-processed against the genetic phenotyping model (Figure S6). In all the cases, the correct (ER–/PR-HER−) phenotype was indicated by the model.

These findings clearly show that the recognition models generated by our robust single-cell analytical MSI workflow are relevant for the identification of cell subtypes in a tissue environment. Moreover, our method provides detailed spatial information in an automatic and thus objective manner on where these subtypes are present in the tissue, highlighting the enormous clinical potential of the method. Indeed, as described above, genetic phenotyping is linked to diagnostic and therapy prognostics. Being able to identify the correct breast cancer phenotype in a fast and objective manner without generally known staining disadvantages will significantly improve diagnosis speed and accuracy, leading to better patient treatment and outcome.

### Toward Automated Online Contextual Cell Type Recognition

As addressed in the fourth criteria of the workflow, ideally the subtype identification based on the developed recognition models can be applied “on-the-fly”, that is, during the imaging run. This would save post-processing time, and intraoperative diagnostics would become possible. To move toward online “on the spot” recognition, we need an MALDI system that is compatible with online recognition software. Unfortunately, timsTOF fleX is not (yet) compatible with any online recognition software for on-demand measurements. However, because we showed that Synapt G2-Si HDMS results in similar molecular single-cell profiles, we expect that both instruments, measuring the same sample and using the same recognition model, will result in the same identified cell subtypes. To verify this hypothesis, we first performed measurements with both instruments on a MDA-MB-231 xenograft sample with 30 × 30 μm^2^ spatial resolution. Post-processing was performed on both analyses (SCiLS Lab for timsTOF flex and AMX recognition for Synapt G2-Si HDMS), resulting in the correctly identified main cell subtype MDA-MB-231 with comparable percentages (64.71%—timsTOF and 65.94%—Synapt) (Figure 6). These percentages are the % of pixels (100% is the full measured region/area) classified as the indicated cell type. Meaning that about 65% of the full tissue is recognized as being MDA-MB-231. The rest of the tissue is necrotic area, thus recognized as an outlier, CAL 120 or MCF7. This corresponds with the information that is given by the pathologist based on the H&E staining (necrotic areas indicated in Figure 6). Other reported cell types (CAL 120 and MCF-7) by SCiLS Lab can be seen as errors related to the use of the SCiLS Lab program itself. Indeed, unlike SCiLS Lab, AMX recognition software allows outliers to be classified, for which 32.42% were assigned. It should be noted that this rate is very similar to the total misclassified cell subtypes (35.29%) reported by SCiLS Lab and is related to the necrotic regions present in the sample.

Taking these into consideration, these results confirm once again our earlier findings that the same recognition model is applicable independently of the used MALDI instrument and that the percentage of the main recognized cell type is independent of the used offline recognition program (SCiLS Lab or AMX recognition).

Finally, to test the real-time “on the spot” recognition capability of our developed system, our AMX subtype recognition model was loaded into AMX online recognition software and MDA-MB-231 xenografts were analyzed on Synapt G2-Si HDMS at a 30 × 30 μm^2^ pixel size. As shown in the video (Figure S7), we were able to correctly identify the correct cell subtype for every single laser spot within a second. These data demonstrate that on-demand breast cancer cell recognition using MSI can be achieved for digital pathology. This fully automated recognition system is objective, does not require any prior staining or labeling, and identifies single cells much faster than any other pathological diagnosis system on the market.

## Conclusions

We developed a robust method to conduct imaging MS at the subcellular level and extract molecular profiles of cultured single individual cells. We were able to investigate subtype heterogeneity and identify 79 different lipids present in different ratios in all 14 cultured breast cancer cell subtypes. These subtype-specific molecular profiles were implemented in cell subtype recognition models and their tissue relevance was successfully shown. Our repeatable imaging method opens the possibility to discover molecular differences at a single-cell level, including inter- and intracellular processes and alterations. This represents major opportunities for basic research (understanding the origin and development of cancer) and clinical diagnostics and prognostics.

Our model was generated based on the full mass spectrum, which means that other compound classes, including potential biomarkers, may have contributed to the excellent cross-validation results. The presented models can be utilized as research tools for the pathway and biomarker discovery, as they can pinpoint significant differences in molecular profiles. Future studies will focus on the further optimization of these subcellular molecular imaging possibilities, for example, by increasing spatial resolution and sensitivity and targeting intracellular pathways. Our presented methodology opens up research possibilities that investigate changes in intracellular pathways as a consequence of diseases and therapy responses. This will lead to a better understanding of diseases and more pathway-focused therapy development. Indeed, when visualizing single cells and directly identifying them in a tissue environment, their molecular behavior and interaction in the context of a (disease-related) changing tissue environment can be studied.

This contextual single-cell recognition and cell typing, with MSI and recognition models, also offers several advantages over traditional diagnostic and prognostic approaches for breast cancer. First, it rapidly detects ER, PR, and HER2 status without using labeling techniques or antibody stains. Moreover, the presented MALDI-MSI approach is automated, and therefore much less susceptible to technical variance (e.g., fixation time in IHC) and subjective scoring and interpretation. This newly developed approach enables objective analyses that could directly lead to better patient care. MALDI-MSI-based cell recognition also opens opportunities for studying the effects of new treatments on tumor heterogeneity, possibly facilitating personalized medicine in the near future. Future studies that apply our method and develop single-cell databases of other cancer-associated cell lines could significantly enhance our knowledge and insight into compound distributions and cell–cell interactions.

The presented MALDI-MSI-based cancer cell recognition model fulfills the main requirements for digital pathology: (1) robustness, repeatability, and independence of the instrument platform; (2) speed; and (3) clear and objective interpretation without the need for in-depth MS knowledge. Furthermore, it complements time-consuming IHC tests in the digital pathology toolbox. The potential of MSI to be integrated routinely into digital pathology workflows will increase as vendor-independent data analysis software, including model builders and online recognition software, becomes available.^24^

## Supporting Information Available

Additional materials and methods detail on the used cell lines and additional figures as mentioned in the text (PDF)
Additional video as mentioned in the text of online recognition during MALDI imaging run, human breast cancer cell lines and cell culture conditions, single-cell MALDI-TOF images, optical microscopy images, cellular distribution PC 36:1 and LPC 18:0, comparison single-cell mass spectra cell lines, comparison mass spectra different instruments, comparison mass spectra cell images and cell pellets, cross-validation recognition model, intensity box plots lipids, classification breast cancer xenograft, and video online recognition (MOV)

## Supplementary Material

ac1c05238_si_001.pdf

ac1c05238_si_002.mov
