# Supplementary material for: ‘On the Spot’ Digital Pathology of Breast Cancer Based on Single-Cell Mass Spectrometry Imaging
Source: Anal Chem. 2022 Apr 12;94(16):6180–90. doi: 10.1021/acs.analchem.1c05238 (PMC9047448; doi:10.1021/acs.analchem.1c05238)
Supplement: Supplementary file 1 — ac1c05238_si_001.pdf [file ac1c05238_si_001.pdf]

Supporting information

‘On the spot’ digital pathology of breast cancer based on a single-cell mass spectrometry imaging

Eva Cuypers[a]*, Britt S.R. Claes[a], Rianne G. Biemans[b], Natasja Lieuwes[b], Kristine Glunde[c,d], Ludwig Dubois[b], Ron M.A. Heeren[a]

[a] Dr. E Cuypers, B.S.R. Claes, Prof. Dr. R.M.A. Heeren
Maastricht MultiModal Molecular Imaging Institute (M4i), Division of Imaging Mass Spectrometry,
University of Maastricht
Universiteitssingel 50, 6229 ER Maastricht,The Netherlands

[b] R. Biemans, N. Lieuwes, Prof. Dr. L. Dubois
The M-Lab, Department of Precision Medicine, GROW – School for Oncology,
University of Maastricht
Universiteitssingel 50, 6229 ER Maastricht, (The Netherlands

[c] Prof. Dr. K. Glunde
Russell H. Morgan Department of Radiology and Radiological Science, Division of Cancer Imaging Research,
The Johns Hopkins University School of Medicine, Baltimore, MD, USA;

[d] Prof. Dr. K. Glunde
The Sidney Kimmel Comprehensive Cancer Center,
The Johns Hopkins University School of Medicine, Baltimore, MD, USA

* contact information corresponding author: Dr. Eva Cuypers, Universiteitssingel 50, 6229 ER Maastricht, The Netherlands, Phone: +31 43 388 1501, email: e.cuypers@maastrichtuniversity.nl, webpage: www.maastrichtuniversity.nl/M4I

Contents

Table S1: Human breast cancer cell lines used in this study and cell culture conditions used. 2

Figure S1: Single-cell images of 4 different breast cancer cell lines measured by MALDI-TOF imaging with a spatial resolution of 5 × 5 μm^2. 3

Figure S2: Comparison of single cell mass spectra (*m/z* 700-850, region of most common lipids shown) for two different cell cultures per cell subtype. Measurements were performed using timsTOF fleX MALDI-2. 4

Figure S3: A. Comparison of mass spectra obtained with TimsTOF fleX (MALDI-1, positive mode) from mass spectrometry imaging (red) and pellet profile (green). B. Bar plot of the mean normalized mass profiles obtained with MSI and from the cell pellet of 8 differentiating lipids of single MDA-MB-231 and HCC 1143 cells after RMS normalization. 5

Figure S5: Intensity box plots of the top 10 differentiating lipids in representative cell ROIs............................... 8

Figure S6: Classification example of breast cancer xenograft MDA-MB-231 according to ER, PR, HER2 status. 9

Figure S7: Video online recognition on xenograft sample (separate video file)... 10

Cell line	Origin	Code (Povider)	Povider	Medium to culture cells at CP	ER	PR	HER2	BRCA1	Subtype	Tumor
CAL-120	human	ACC 459	DSMZ	90% DMEM + 10% FBS	-	-	-	WT	TNB	AC
EFM-19	human	ACC 231	DSMZ	90% RPMI 1640 + 10% FBS	+	+	-	WT	LA	IDC
HCC-1143	human	ACC 517	DSMZ	80% RPMI + 20% FBS	-	-	-	WT	TNA	DC
HCC-1937	human	ACC-513	DSMZ	90% RPMI 1640 + 10% FBS	-	-	-	MU	TNA	DC
HCC-1954	human	CRL-2338	ATCC	90% RPMI 1640 + 10% FBS	-	-	+	WT	H	DC
HDQ-P1	human	ACC 494	DSMZ	90% DMEM + 10% FBS	-	-	-	MU	TNB	IDC
HS-578T	human	ACC 781	DSMZ	90% DMEM + 10% FBS	-	-	-	WT	TNB	IDC
JIMT-1	human	ACC 589	DSMZ	90% DMEM + 10% FBS	-	-	+	WT	H	DC
MDA-MB-436	human	HTB-130	ATCC	90% DMEM + 10% FBS	-	-	-	MU	TNA	AC
MDA-MB-231	human	ACC-732	DSMZ	90% DMEM + 10% FBS	-	-	-	WT	TNB	AC
MDA-MB-453	human	ACC-65	DSMZ	90% DMEM + 10% FBS	-	-	+	WT	H	AC
MDA-MB-468	human	ACC-738	DSMZ	90% DMEM + 10% FBS	-	-	-	WT	TNA	AC
MCF-7	human	HTB-22	ATCC	90% DMEM + 10% FBS	+	+	-	WT	LA	IDC
MCF10A	human	CRL-10317	ATCC	90% DMEM + 10% FBS	-	-	-	WT	luminal ductal gland	epithelium

ER=oestrogen receptor
PR=progesteron receptor
HER=human epidermal growth factor receptor 2

WT=wildtype
ND=not decided
MU=mutated

L=luminal
H=Her2

IDC=invasive ductal carcinoma
AC=adenocarcinoma
DC=ductal carcinoma
InfDC=inflammatory ductal carcinoma

Table S1: Human breast cancer cell lines used in this study and cell culture conditions used.

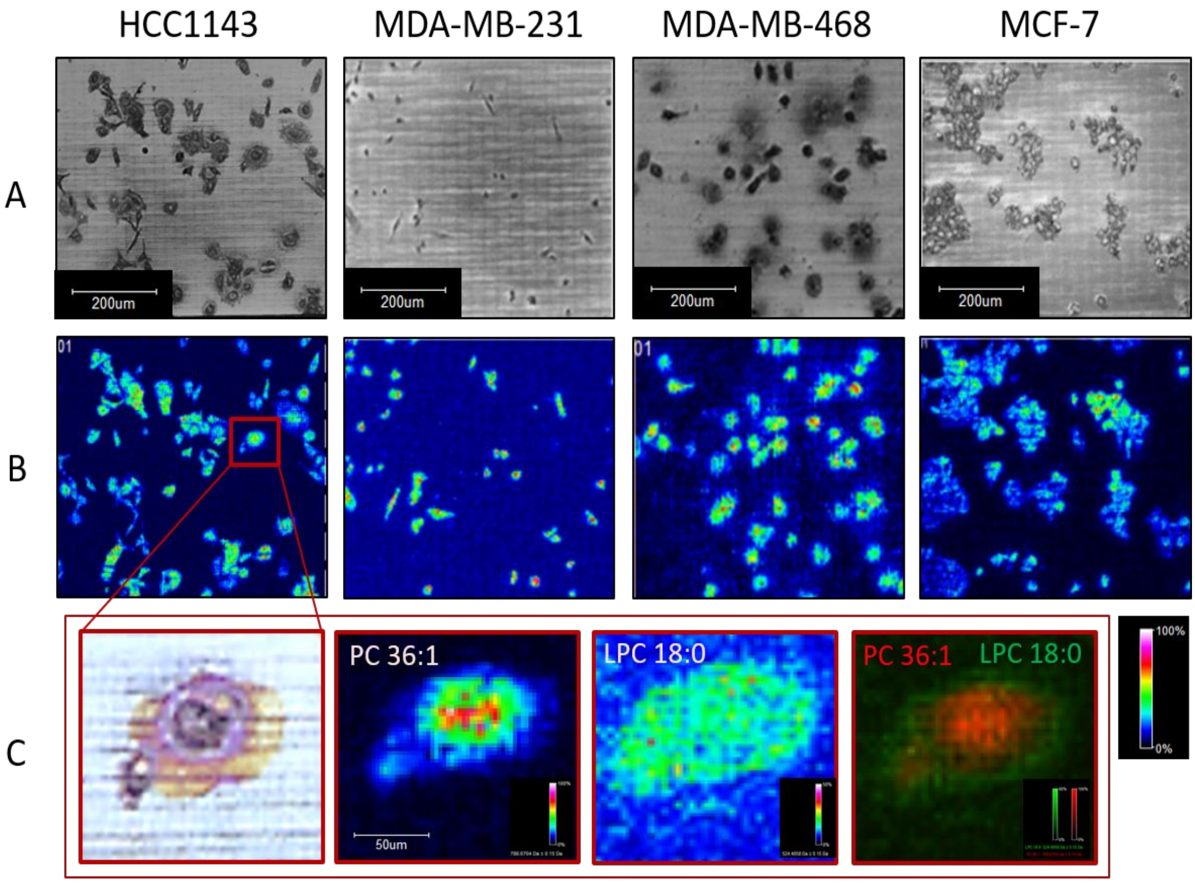

Figure S1: Single-cell images of 4 different breast cancer cell lines measured by MALDI-TOF imaging with a spatial resolution of 5 × 5 µm².

A. Optical microscopy images (10×) acquired following mass spectrometry imaging. **B.** Distribution of PC 36:1 in the different breast cancer cell lines. **C.** Magnified images of a single HCC1143 cell (inset). Optical image (left), spatial distributions of PC 36:1 (middle left), LPC 18:0 (middle right), and their merged image (right). Scale bars in A represent 200 µm and apply also to B; scale bar in C represents 50 µm.

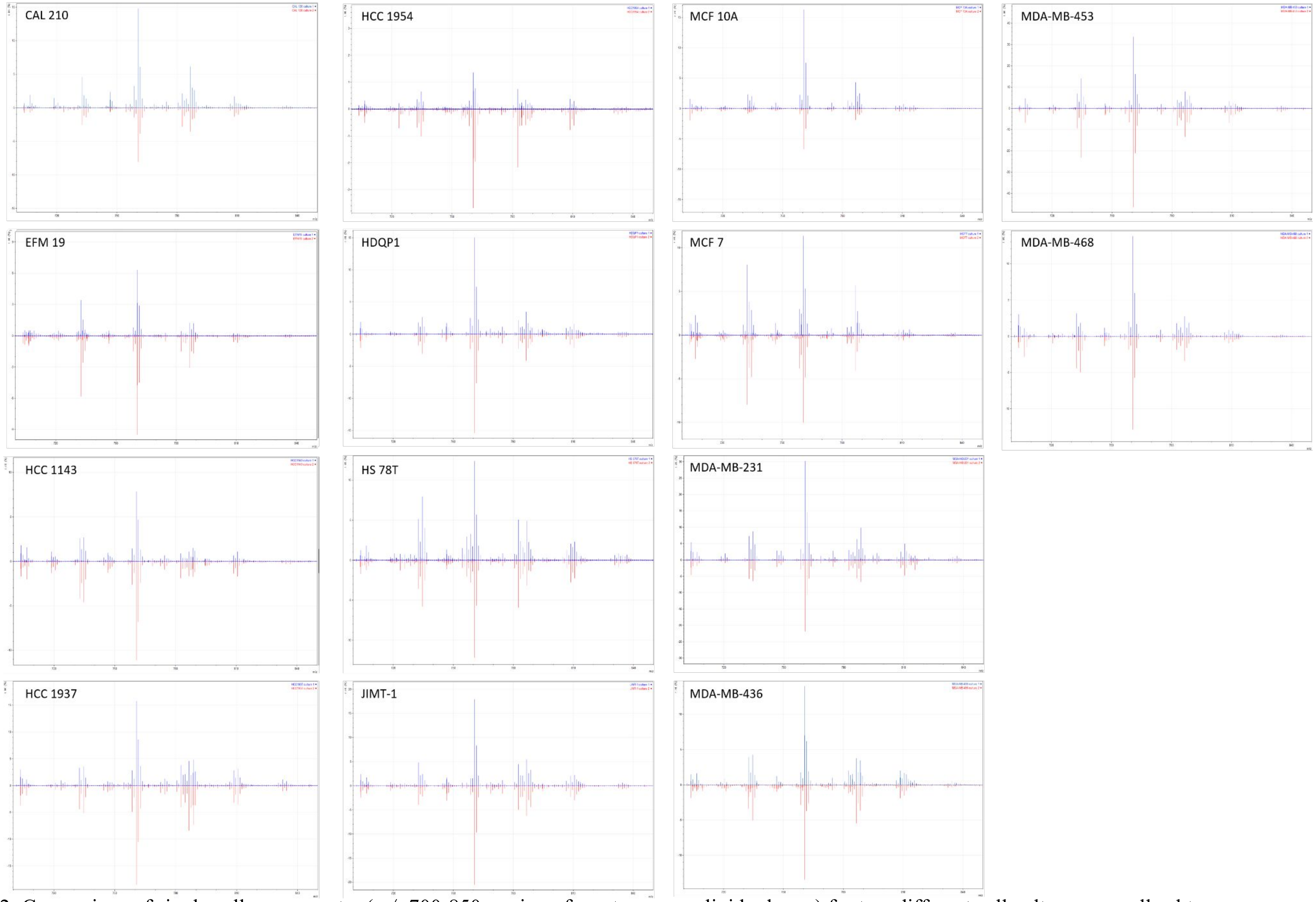

Figure S2: Comparison of single cell mass spectra (*m/z* 700-850, region of most common lipids shown) for two different cell cultures per cell subtype. Measurements were performed using timsTOF fleX MALDI-2.

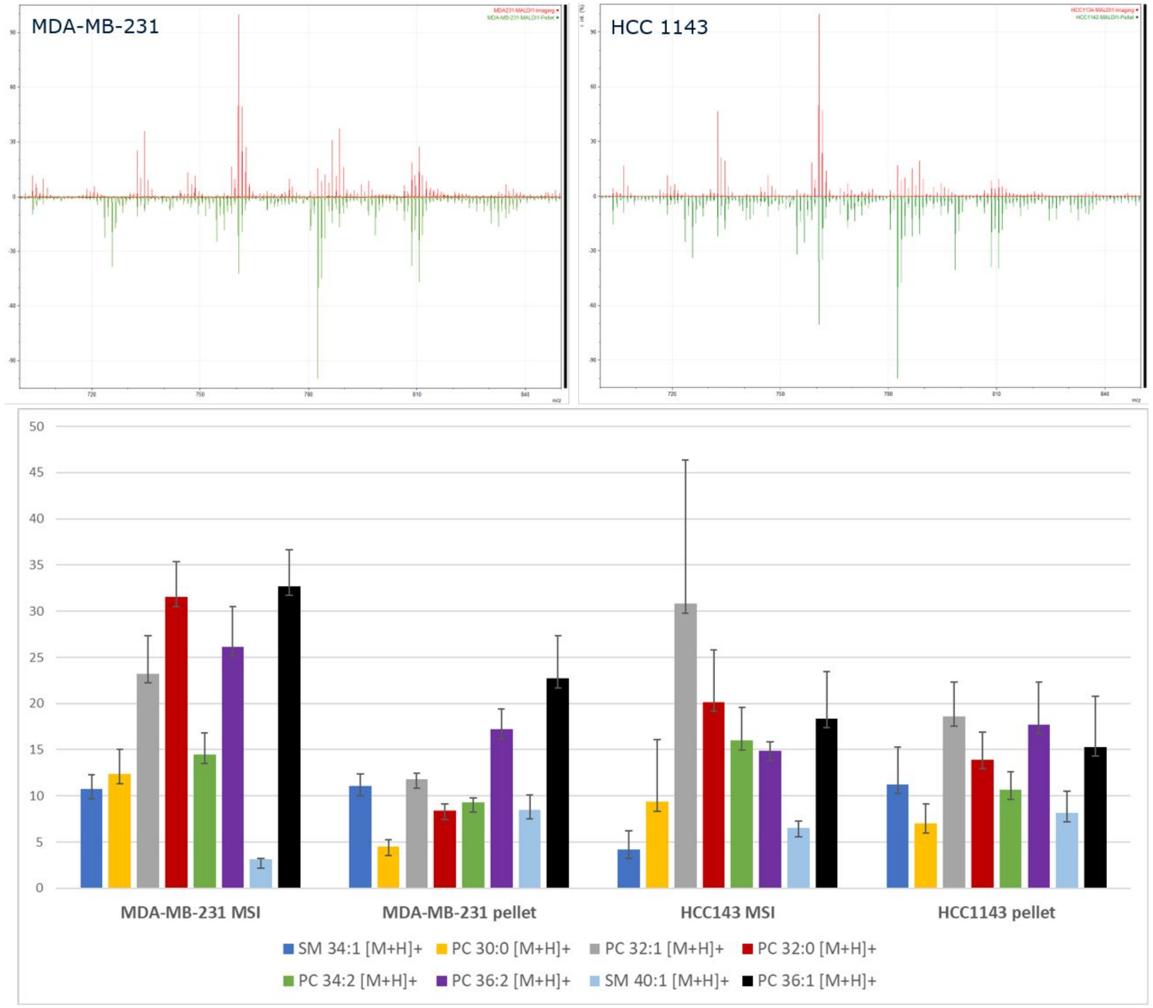

Figure S3: A. Comparison of mass spectra obtained with TimsTOF fleX (MALDI-1, positive mode) from mass spectrometry imaging (red) and pellet profile (green). B. Bar plot of the mean normalized mass profiles obtained with MSI and from the cell pellet of 8 differentiating lipids of single MDA-MB-231 and HCC 1143 cells after RMS normalization.

Error bars represent standard deviations from three randomly chosen cell profiles or pellets. A significant difference is found in lipid ratios between MSI and pellet based mass spectra.

A

Validation Type	20% out
Model type	Lda
Number of Pca dimensions	25
Number of Lda dimensions	2
Outlier Type	Based on standard deviation
Parameter	3

Group result:

Group	Number of spectra	Number of passes	Number of failures	Number of outliers	Correct Classification Rate	
					Excluding outliers	Including outliers
Total	229	203	13	13	93.98%	88.65%

Confusion matrix:

	er-, pr-, her-	er-, pr-, her+	er+, pr+, her -	Outlier	Total
er-, pr-, her-	143	12	0	9	164
er-, pr-, her+	1	31	0	1	33
er+, pr+, her -	0	0	29	3	32
Total	144	43	29	13	229

B

Validation Type	20% out
Model type	Lda
Number of Pca dimensions	12
Number of Lda dimensions	7
Outlier Type	Based on standard deviation
Parameter	3

Group result:

Group	Number of spectra	Number of passes	Number of failures	Number of outliers	Correct Classification Rate	
					Excluding outliers	Including outliers
Total	227	198	6	23	97.06%	87.22%

Confusion matrix:

	cal 120	efm 19	hcc 1143	hcc 1937	hcc 1954	hdqp1	hs 578t	jimt-1	mcf 10a	mcf 7	mda-mb-231	mda-mb-436	mda-mb-453	mda-mb-468	Outlier	Total
cal 120	17	0	0	0	0	0	0	4	0	0	0	0	0	0	0	21
efm 19	0	15	0	0	0	0	0	0	0	0	0	0	0	0	2	17
hcc 1143	0	0	16	0	0	0	0	0	0	0	0	0	0	0	1	17
hcc 1937	0	0	0	17	0	0	0	0	0	0	0	0	0	0	0	17
hcc 1954	0	0	0	0	14	0	0	0	0	0	0	0	0	0	2	16
hdqp1	0	0	0	0	0	11	0	0	0	0	0	0	0	0	4	15
hs 578t	0	0	0	0	0	0	19	0	0	0	0	0	0	0	3	22
jimt-1	0	0	0	0	0	0	0	17	0	0	0	0	0	0	0	17
mcf 10a	0	0	0	0	0	0	0	0	8	0	0	0	0	0	4	12
mcf 7	0	0	0	0	0	0	0	0	0	14	0	0	0	0	1	15
mda-mb-231	0	0	0	0	0	0	0	0	0	0	15	0	0	0	1	16
mda-mb-436	0	0	0	0	0	0	0	0	0	0	0	15	0	0	2	17
mda-mb-453	0	0	0	0	0	0	0	0	0	0	0	0	7	0	2	9
mda-mb-468	0	0	0	0	0	0	0	0	1	0	0	1	0	13	1	16
Total	17	15	16	17	14	11	19	21	9	14	15	16	7	13	23	227

Figure S4: Detailed cross-validation table of **A.** different breast cancer subtypes based on ER, PR, HER2 status and **B.** breast cancer cell lines

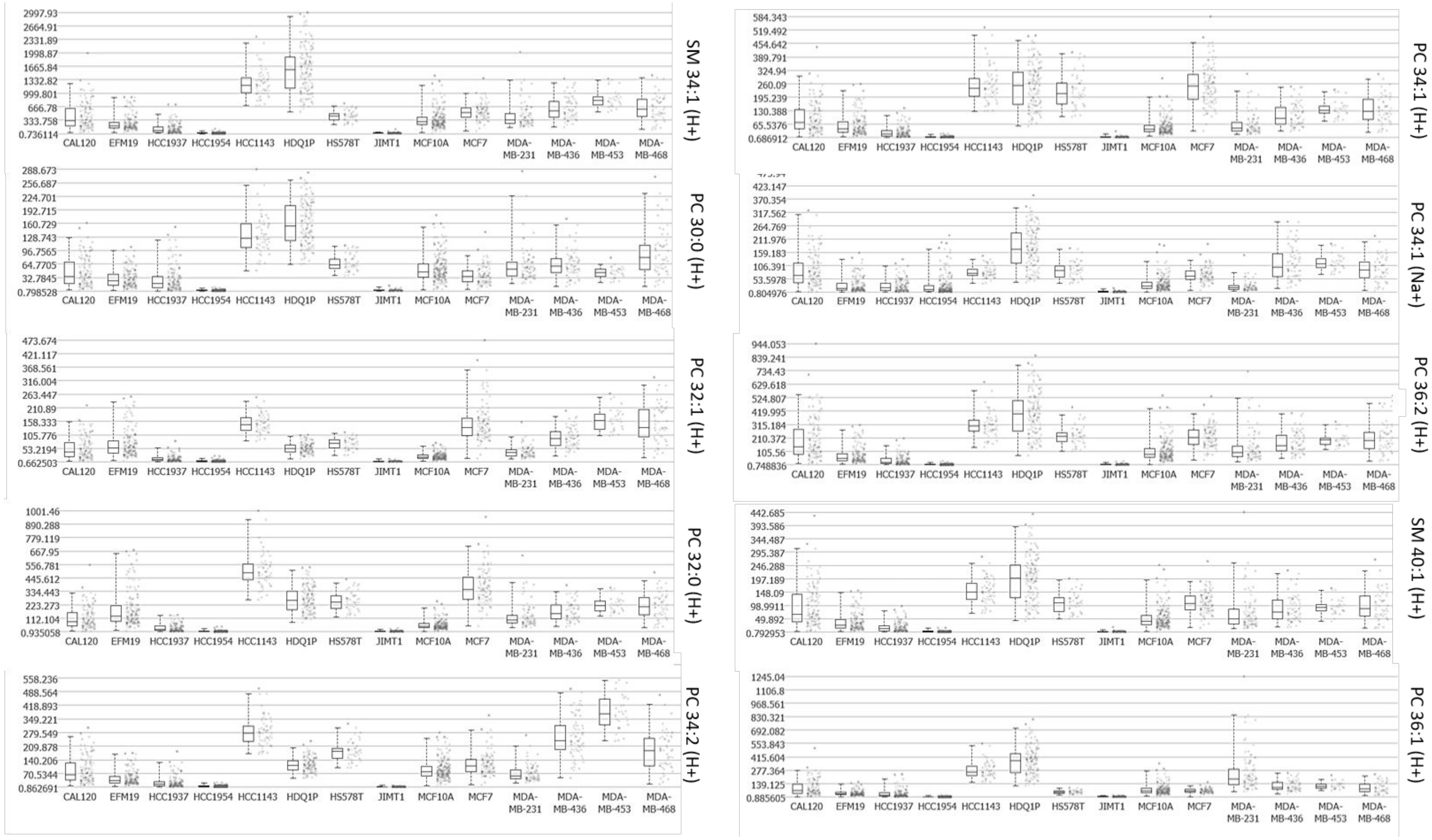

Figure S5: Intensity box plots of the top 10 differentiating lipids in representative cell ROIs.

Error bars represent the variance detected within the ROI area across the cells analyzed. Each dot next to a box-and-whisker represents the intensity of a single pixel scan.

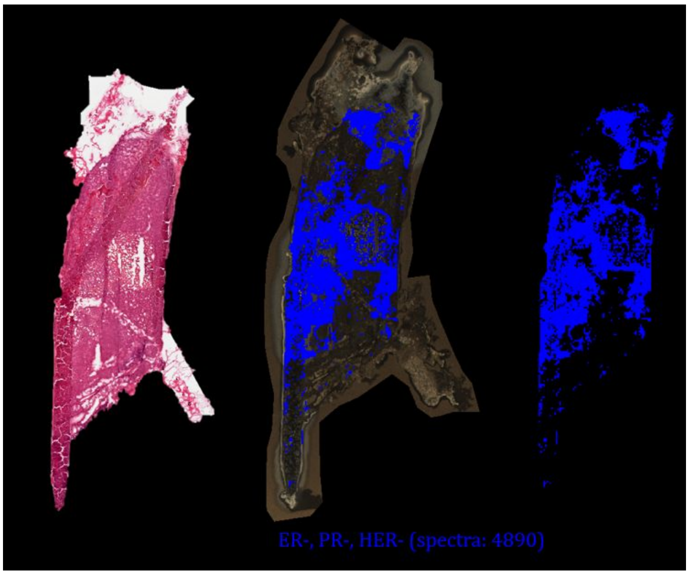

Figure S6: Classification example of breast cancer xenograft MDA-MB-231 according to ER, PR, HER2 status.

Left: H&E staining; Middle: overlay visual scan of the non-stained section prior to IMS and identified genetic phenotype status; right: identified genetic phenotype status.

Figure S7: Video online recognition on xenograft sample (separate video file)

An MDA-MB-231 breast tumor xenograft section was measured with Synapt G2-Si HDMS in positive ion mode at 1 scan per second. AMX software with our recognition model was loaded. In every scan, the cell line is identified 'on-the-fly'. When the cell was not recognized as a particular line, the system output designated 'outlier.' The cell line was correctly identified in the tissue and the outliers correctly denoted. Remark: the xenograft in this video was taken from a different tissue (from another animal) as that shown in Figures 6 and S6.
